# Supplementary material for: MDPath: Unraveling Allosteric Communication Paths of Drug Targets through Molecular Dynamics Simulations
Source: J Chem Inf Model. 2025 Oct 1;65(20):11123–35. doi: 10.1021/acs.jcim.5c01590 (PMC12570139; doi:10.1021/acs.jcim.5c01590)
Supplement: Supplementary file 1 [file ci5c01590_si_001.pdf]

Supplementary Information for "MDPath: Unraveling Allosteric Communication Paths of Drug Targets through Molecular Dynamics Simulations"

Niklas Piet Doering,[†,‡] Marvin Taterra,[†,¶] Marcel Bermúdez,[*,¶] and Gerhard Wolber[*,‡]

†*equally contributed to this work*

‡*Department of Biology, Chemistry and Pharmacy, Institute of Pharmacy, Molecular Design Group, Freie Universität Berlin, Königin-Luisestr. 2+4, 14195 Berlin, Germany*

¶*Department of Theoretical Chemistry, Institute of Pharmaceutical and Medicinal Chemistry, Universität Münster, Correnstr. 48, 48149, Münster, Germany*

E-mail: m.bermudez@uni-muenster.de; gerhard.wolber@fu-berlin.de

Phone: +49 251 83 32272; +49 30 838 52686. Fax: +49 251 83 32211; +49 30 838 452686

Supplementary Information Contents

Dynophore Analysis **3**

Salbutamol 3

Carazolol 5

DAMGO 7

Effects of ICL3 Truncation **8**

Bootstrapping Analysis **9**

Effects of the Tertiary Complex on GPCR Activation **11**

Full Allosteric Paths **15**

Effects of Allosteric Modulators (Sodium Ions and Cholesterol) **16**

List of Supplementary Information Figures **19**

List of Supplementary Information Tables **20**

References **21**

Dynophore Analysis

Salbutamol

Table S1: Most frequent receptor-ligand interactions between salbutamol and the β_2-adrenoceptor identified by *Dynophores*.[1–6] Key binding site residues and interaction types are listed, highlighting the dynamic nature of these interactions.

**GPCR Residues**	**Interaction Types**
$V114^{3.33}$, $V117^{3.36}$	Hydrophobic
$F290^{6.52}$	
$S203^{5.42}$, $S207^{5.46}$	H-bond acceptors
$N312^{7.39}$	
$D113^{3.32}$, $S203^{5.42}$	H-bond donors
$S207^{5.46}$,$N312^{7.39}$	
$D113^{3.32}$	Positive ionizable

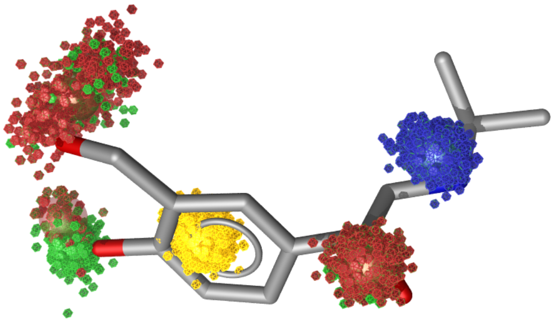

Figure S1: Point cloud representation of the salbutamol dynophore. We can see concise point clouds proving the stability of the ligand during the MD simulation. Lightly distorted clouds are seen for the phenolic hydroxy groups, as these switch their protein interaction partners between residues S203$^{5.42}$, S204$^{5.43}$ and S207$^{5.46}$. Each point within a cloud represents an interaction in one frame, with blue indicating positive ionizable interaction, red hydrogen bond acceptors, green hydrogen bond donors and yellow hydrophobic contacts.

Carazolol

Table S2: Summary of the most frequent receptor-ligand interactions between carazolol and the β_2-adrenoceptor identified by *Dynophores*.[1–6] Key binding site residues and interaction types are listed, highlighting the dynamic nature of these interactions.

**GPCR Residues**	**Interaction Types**
$V114^{3.33}$, $V117^{3.36}$	Hydrophobic
$F193^{ECL2}$, $T195^{ECL2}$	
$A200^{5.39}$, $F290^{6.52}$	
$N312^{7.39}$, $Y316^{7.43}$	H-bond acceptors
$D113^{3.32}$, $S203^{5.42}$	H-bond donors
$N312^{7.39}$	
$D113^{3.32}$	Positive ionizable

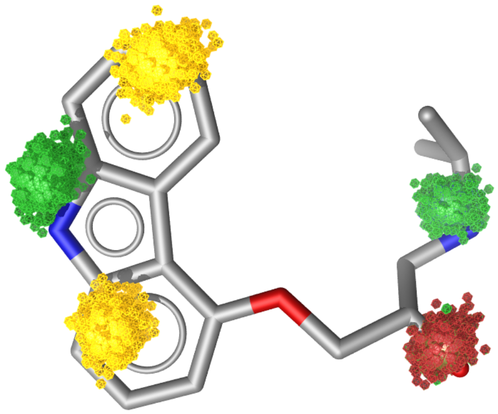

Figure S2: Point cloud representation of the carazolol dynophore. We can see concise point clouds proving the stability of the ligand during the MD simulation. Each point within a cloud represents an interaction in one frame, with blue indicating positive ionizable interaction, red hydrogen bond acceptors, green hydrogen bond donors and yellow hydrophobic contacts.

DAMGO

Table S3: Summary of receptor-ligand and receptor-G protein interactions identified and tracked using *Dynophores*.[1–6] Key binding site residues and interaction types are listed for each ligand, highlighting the dynamic nature of these interactions.

**Ligand**	**GPCR Residues**	**Interaction Types**
DAMGO	W135^{ECL1}, V145$^{3.28}$, I146$^{3.29}$ Y150$^{3.33}$, M153$^{3.36}$, I298$^{6.51}$ V302$^{6.55}$, I324$^{7.39}$	Hydrophobic
	Q129$^{2.60}$, H299$^{6.52}$, W320$^{7.35}$	H-bond acceptors
	Q126$^{2.60}$, D149$^{3.32}$, C219^{ECL2} E231$^{5.35}$, Y328$^{7.43}$	H-bond donors
	D149$^{3.32}$	Positive ionizable
G_i-protein	V171$^{3.54}$, L178^{ICL2}, M257$^{5.61}$ L261$^{5.65}$, V264$^{5.68}$, I280$^{6.33}$ V284$^{6.37}$	Hydrophobic
	T99$^{12.48}$, R181^{ICL2}, R279$^{6.32}$ E343$^{8.48}$, R347$^{8.52}$	H-bond acceptors
	A170$^{3.53}$, P174^{ICL2},I258$^{5.62}$ R260$^{5.64}$, K262$^{5.66}$	H-bond donors
	R181^{ICL2}, R265^{ICL3}	Negative ionizable
	W194$^{4.50}$	Positive ionizable

Effects of ICL3 Truncation

The truncation of ICL3, as observed in several *in silico* β2-adrenoceptor studies,[7,8] produced similar signaling pathways at the binding site but struggled to replicate signals towards the intracellular regions. In our analysis of a truncated β_2-adrenoceptor in complex with salbutamol, we observe overall less defined signaling pathways. Pathways that are typically seen in activation like the connection between I3.40 to W6.48 PIW motive are still present, however the closer paths come to the truncated ICL region the more paths we see that are not linked to activation. For example we can now clearly see strong correlations of the D and R of the DRY motive while no connection to the Y is seen. This pattern would typically be indicative of inactivation and is seen in the carazolol case. During visual inspection of the truncated simulation larger movements at the truncated helix tips were observed, which may significantly affecting the NMI with residues at the intracellular side of TM5 and 6. Therefore, we conclude that meticulous modeling of the full system is crucial for accurate analysis with *MDPath*.

Bootstrapping Analysis

Table S4: Standard errors within top 500 paths based on 500 bootstrap samples for each base analysis.

**System**	**Simulation 1**	**Simulation 2**	**Simulation 3**
Active β2-adrenoceptor + salbutamol	1.932	2.805	1.997
Inactive β2-adrenoceptor + carazolol	1.409	1.471	1.120
Active A2A receptor+ adenosine	1.471	2.396	2.617
Inactive A2A receptor + caffeine	2.029	1.408	1.217
Inactive A2A receptor + caffeine + cholesterol + sodium ion	1.120	1.490	1.222
Inactive MOR + alvimopan	1.148	1.435	0.891
Active MOR + DAMGO + G_i-protein	1.636	1.486	1.307
ABL1 Kinase + Ascinimib + other kinase	1.145	1.776	1.382

Table S5: Standard errors within top 500 paths based on 500 bootstrap samples for each ligand-based analysis.

**System**	**Simulation 1**	**Simulation 2**	**Simulation 3**
**Contact Based Paths**			
Active MOR + DAMGO + G_i-protein (DAMGO based)	0.906	0.603	0.956
Active MOR + DAMGO + G_i-protein (G_i-protein based)	1.560	0.786	1.513
Active MOR + DAMGO + G_i-protein (DAMGO and G_i-protein based)	0.424	0.877	1.393
Active β2-adrenoceptor salbutamol (salbutamol based)	0.813	0.212	1.510
Inactive β2-adrenoceptor + carazolol (carazolol based)	0.468	0.075	0.126

Effects of the Tertiary Complex on GPCR Activation

To evaluate how the presence of the G protein influences receptor dynamics, we assessed the activation states of the active-state GPCR simulations through the A100 score.[9] A score above zero indicates active state conformations, while a score below 0 indicated inactive states. In both the tertiary complex (MOR) and the complexes without G protein (β_2-adrenoceptor and A_{2A} receptor), the receptors predominantly remained in conformations consistent with the active state (Figure S3-S5). This indicates that the active state is stable over the course of our simulations. As expected, the presence of the G protein significantly reinforced this stability by further constraining the receptor within the active-state ensemble (Figure S5). Interestingly, in one case, replica 2 of the salbutamol-bound β_2-adrenoceptor, we observed a shift toward slightly more inactive-like conformations towards the end of the simulation (Figure S3), suggesting that while the active state is overall well maintained, subtle deviations can still occur. Thus tracking the activity through means such as the A100 score[9] can greatly aid, when trying to see if the receptor is in a stable state needed for analysis through MDPath.

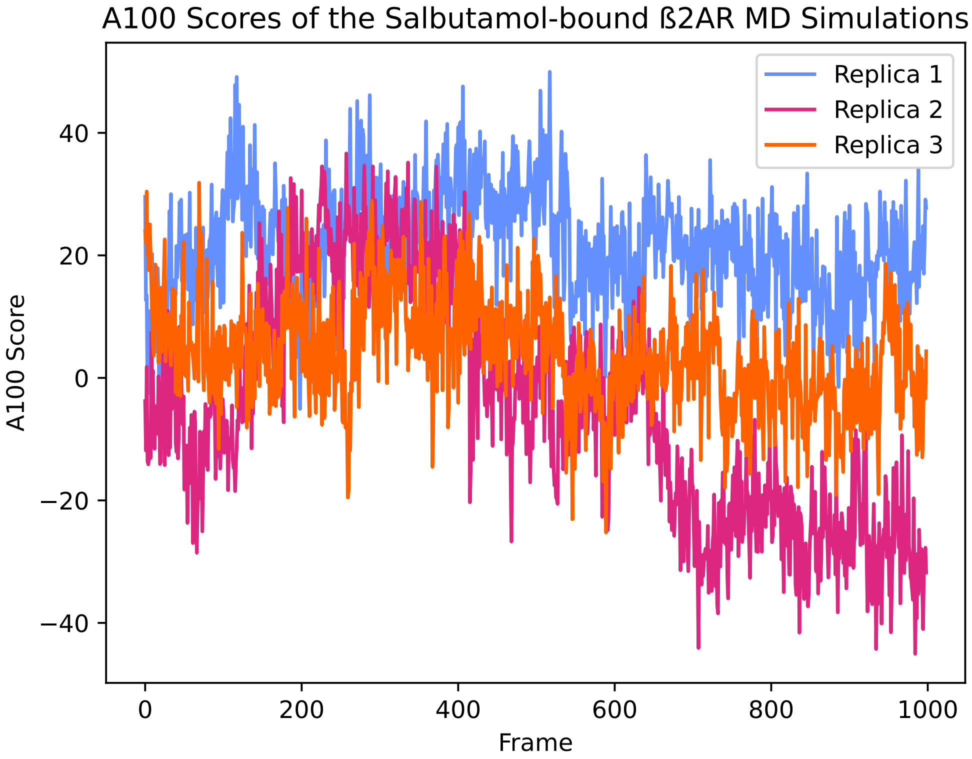

Figure S3: A100 GPCR activation index tracked over the course of the salbutamol-bound β_2-adrenoceptor. It can be seen, that all replicas stay mainly within not inactive conformations ($A100 > 0$). However, a small dip into inactive territory can be seen towards the end of replica 2.

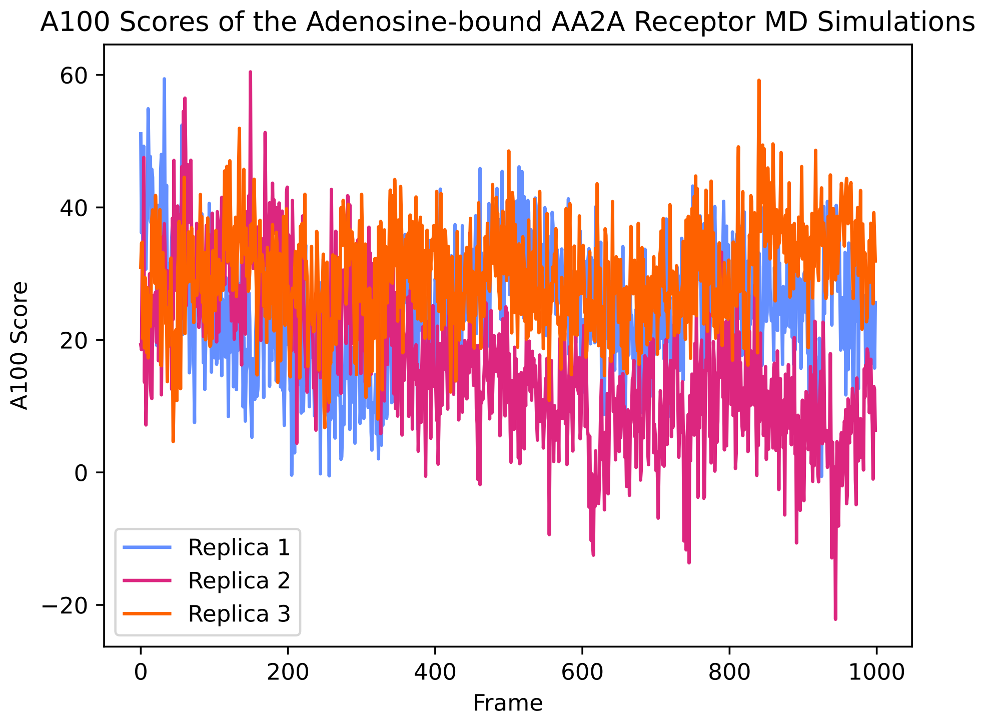

Figure S4: A100 GPCR activation index tracked over the course of the adenosine-bound A_{2A} receptor. It can be seen, that all replicas stay mainly within active conformations ($A100 > 0$).

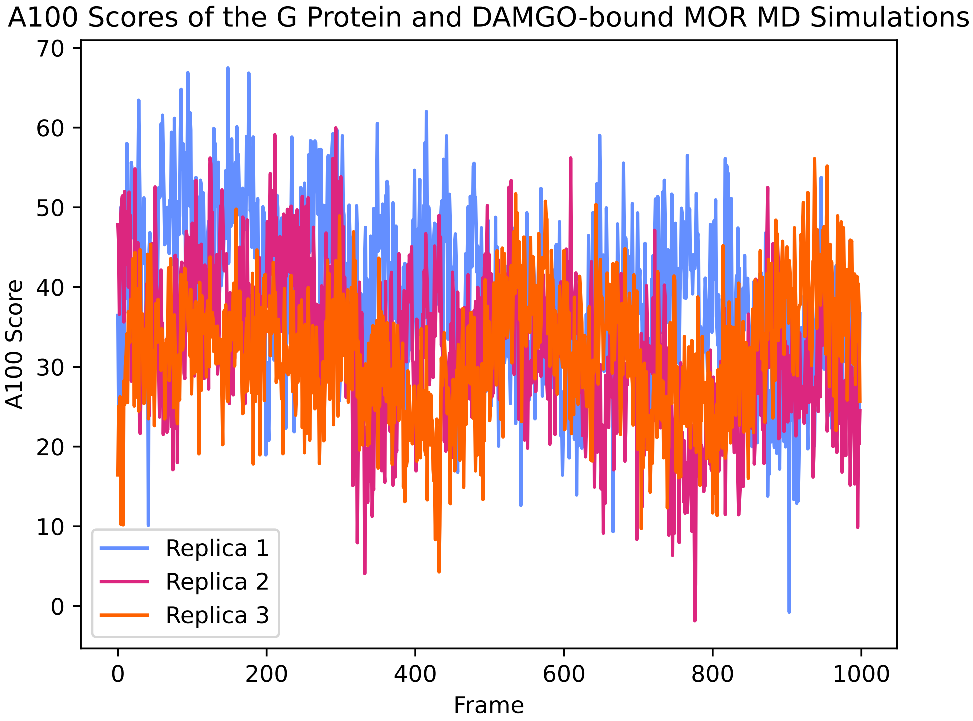

Figure S5: A100 GPCR activation index tracked over the course of the G protein and DAMGO-bound MOR. It can be seen, that all replicas remain thoroughly within active conformations ($A100 > 0$).

Full Allosteric Paths

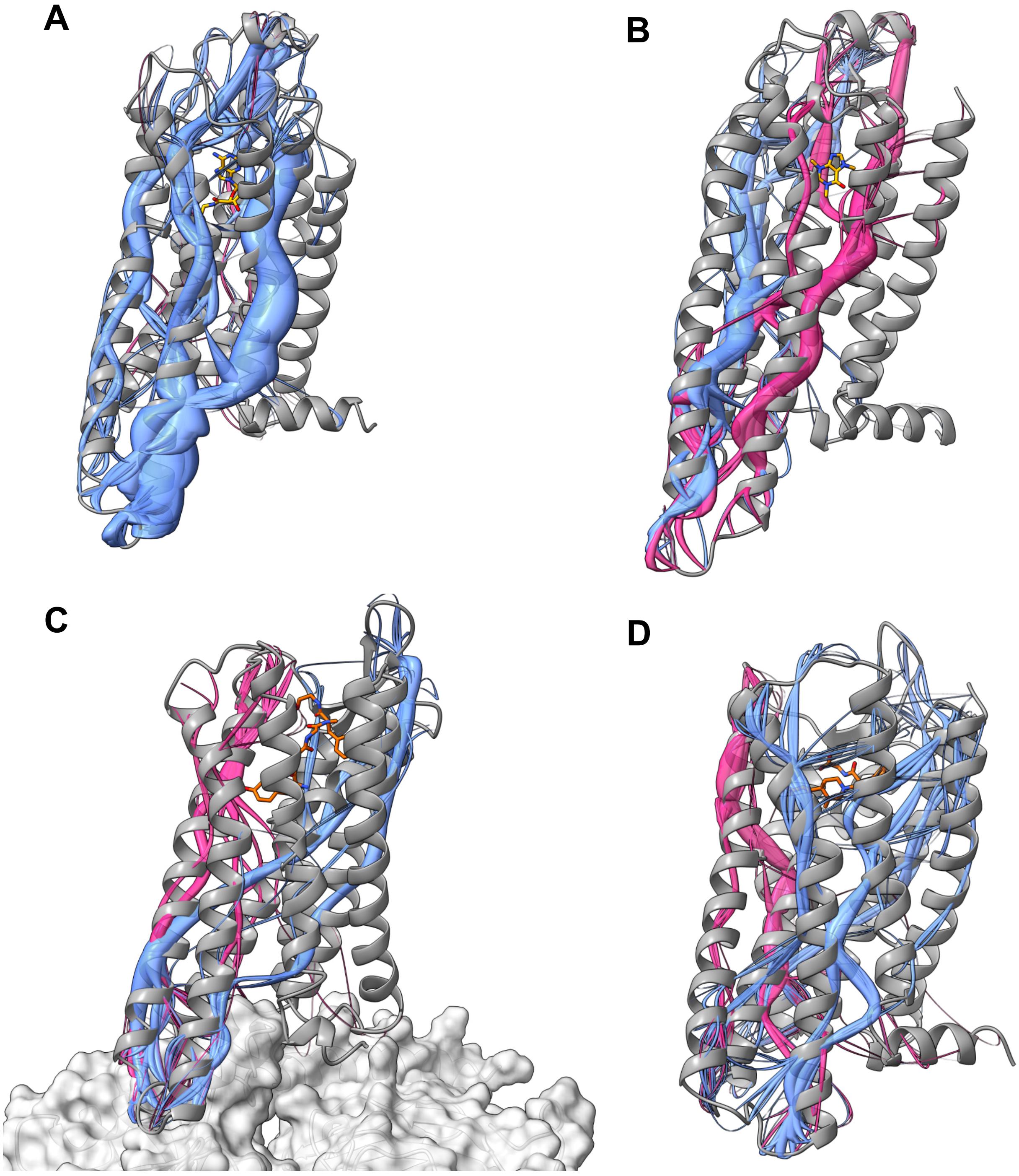

Figure S6: Full allosteric paths of the A_{2A} adenosine receptor and MOR. The respective allosteric paths can be seen in blue and red, while the ligand is shown in yellow. **A.** Full allosteric paths of the active adenosine-bound A_{2A} adenosine receptor. **B.** Full allosteric paths of the inactive caffeine-bound A_{2A} adenosine receptor. **C.** Full allosteric paths of the active G protein and DAMGO-bound MOR. **D.** Full allosteric paths of the inactive alvimopan-bound MOR.

Effects of Allosteric Modulators (Sodium Ions and Cholesterol)

To evaluate the effects of the known allosteric modulators cholesterol and sodium ions, we created a A_{2A} adenosine receptor system where crystalized waters, sodium ions, and cholesterols from the PDB structure were kept (PDB: 5MZP[10]). The rest of the system setup was carried out as discribed in the main text. The simulation setup was done using CharmmGUI..[11] The system was automatically protonated at pH 7.4, additionally, histidine tautomeres were set to $H250^{6.52}$ was protonated at the Nε position, whereas $H264^{ECL3}$ and $H278^{7.43}$ were protonated in both the Nδ and ε position, as discribed in Thomas *et al.*.[12] The system was embedded in POPC lipid bilayer, and solvated with a minimum padding of 10 Å in a cubic TIP3P water box containing 0.15 M NaCl placed automatically. Simulations were performed as specified in the main text.

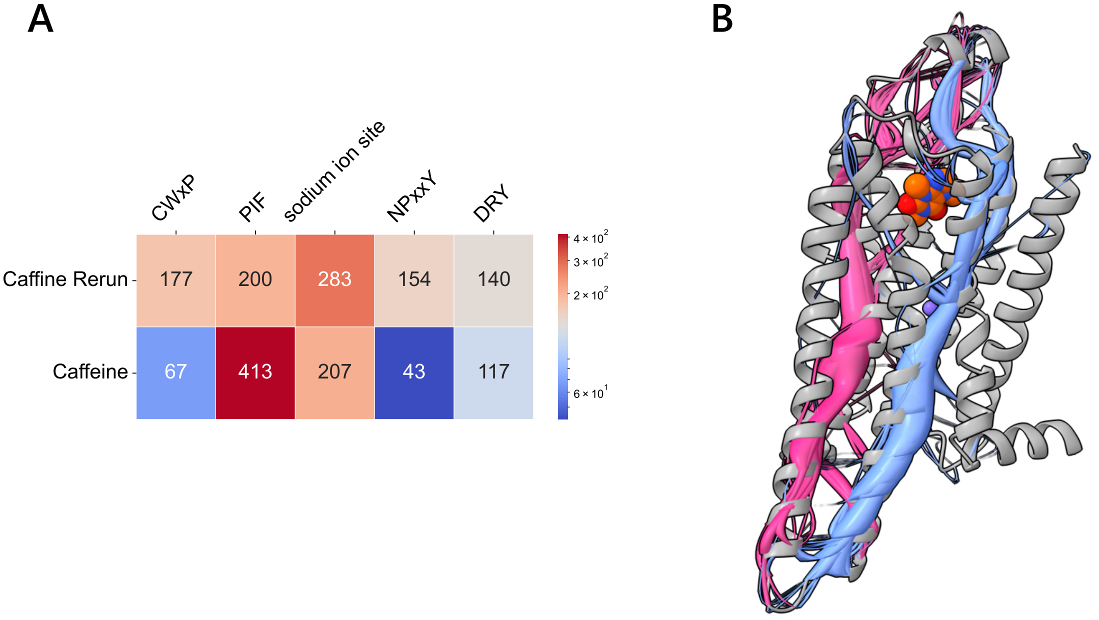

Figure S7: Comparison of alloteric paths identified in the original caffeine simulation and the simulation containing the allosteric modulators cholesterol and sodium (in the sodium ion binding site) **A.** Heatmap showing involvement of conserved class A GPCR motive residues within the top 500 identified paths of all three simulation replicas of the allosteric modulator and caffeine-bound A_{2A} adenosine receptor system, as well as the original system. **B.** Full allosteric paths of the inactiveallosteric modulator and caffeine-bound A_{2A} adenosine receptor. The two alloteric paths are shown in blue and red, caffine depicted in orange, and the sodium ion can be seen as a purple sphere.

MDPath analysis revealed that, while the overall signaling pathways remained largely consistent between the two systems, some differences emerged especially in the weighting of paths (Figure S7). In the model containing additional allosteric modulators, more dominant paths occurred at positions towards the extracellular regions of the GPCR, extending from TM3 toward TM5/TM6. This shift redistributed signaling through both the CWxP and PIF motifs, whereas the simpler system exhibited a stronger preference for PIF-mediated signaling (Figure S7 A). Additionally, we observed increased allosteric communication at the sodium ion site, along with enhanced communication at the adjacent NPxxY motif upon sodium binding. However, the overall pattern of allosteric paths remained rather similar. The calculated standard error of the top 500 paths was also similar. However, the standard

error was slightly lower, showcasing the increased stability caused by the additional allosteric modulators.

List of Supplementary Information Figures

S1 Point cloud representation of the salbutamol dynophore. We can see concise point clouds proving the stability of the ligand during the MD simulation. Lightly distorted clouds are seen for the phenolic hydroxy groups, as these switch their protein interaction partners between residues $S203^{5.42}$, $S204^{5.43}$ and $S207^{5.46}$. Each point within a cloud represents an interaction in one frame, with blue indicating positive ionizable interaction, red hydrogen bond acceptors, green hydrogen bond donors and yellow hydrophobic contacts. . . 4

S2 Point cloud representation of the carazolol dynophore. We can see concise point clouds proving the stability of the ligand during the MD simulation. Each point within a cloud represents an interaction in one frame, with blue indicating positive ionizable interaction, red hydrogen bond acceptors, green hydrogen bond donors and yellow hydrophobic contacts. 6

S3 A100 GPCR activation index tracked over the course of the salbutamol-bound β_2-adrenoceptor. It can be seen, that all replicas stay mainly within not inactive conformations ($A100 > 0$). However, a small dip into inactive territory can be seen towards the end of replica 2. 12

S4 A100 GPCR activation index tracked over the course of the adenosine-bound A_{2A} receptor. It can be seen, that all replicas stay mainly within active conformations ($A100 > 0$). 13

S5 A100 GPCR activation index tracked over the course of the G protein and DAMGO-bound MOR. It can be seen, that all replicas remain thoroughly within active conformations ($A100 > 0$). 14

S6 Full allosteric paths of the A_{2A} adenosine receptor and MOR. The respective allosteric paths can be seen in blue and red, while the ligand is shown in yellow. **A.** Full allosteric paths of the active adenosine-bound A_{2A} adenosine receptor. **B.** Full allosteric paths of the inactive caffeine-bound A_{2A} adenosine receptor. **C.** Full allosteric paths of the active G protein and DAMGO-bound MOR. **D.** Full allosteric paths of the inactive alvimopan-bound MOR. 15

S7 Comparison of alloteric paths identified in the original caffeine simulation and the simulation containing the allosteric modulators cholesterol and sodium (in the sodium ion binding site) **A.** Heatmap showing involvement of conserved class A GPCR motive residues within the top 500 identified paths of all three simulation replicas of the allosteric modulator and caffeine-bound A_{2A} adenosine receptor system, as well as the original system. **B.** Full allosteric paths of the inactiveallosteric modulator and caffeine-bound A_{2A} adenosine receptor. The two alloteric paths are shown in blue and red, caffine depicted in orange, and the sodium ion can be seen as a purple sphere. 17

List of Supplementary Information Tables

S1 Most frequent receptor-ligand interactions between salbutamol and the β_2-adrenoceptor identified by *Dynophores*.[1–6] Key binding site residues and interaction types are listed, highlighting the dynamic nature of these interactions. 3

S2 Summary of the most frequent receptor-ligand interactions between carazolol and the β_2-adrenoceptor identified by *Dynophores*.[1–6] Key binding site residues and interaction types are listed, highlighting the dynamic nature of these interactions. 5

S3 Summary of receptor-ligand and receptor-G protein interactions identified and tracked using *Dynophores*.[1–6] Key binding site residues and interaction types are listed for each ligand, highlighting the dynamic nature of these interactions. 7
S4 Standard errors within top 500 paths based on 500 bootstrap samples for each base analysis. 9
S5 Standard errors within top 500 paths based on 500 bootstrap samples for each ligand-based analysis. 10

References

(1) Sydow, D. Dynophores: Novel Dynamic Pharmacophores. 2015; DOI: 10.18452/14267.

(2) Bock, A.; Bermudez, M.; Krebs, F.; Matera, C.; Chirinda, B.; Sydow, D.; Dallanoce, C.; Holzgrabe, U.; De Amici, M.; Lohse, M. J.; Wolber, G.; Mohr, K. Ligand Binding Ensembles Determine Graded Agonist Efficacies at a G Protein-coupled Receptor. *J. Biol. Chem.* **2016**, *291*, 16375–16389, DOI: 10.1074/jbc.M116.735431.

(3) Wunsch, F.; Nguyen, T. N.; Wolber, G.; Bermudez, M. Structural Determinants of Sphingosine-1-Phosphate Receptor Selectivity. *Arch. Pharm.* **2023**, *356*, 2300387, DOI: 10.1002/ardp.202300387.

(4) Puls, K.; Schmidhammer, H.; Wolber, G.; Spetea, M. Mechanistic Characterization of the Pharmacological Profile of HS-731, a Peripherally Acting Opioid Analgesic, at the Mu-, Delta-, Kappa-Opioid and Nociceptin Receptors. *Molecules* **2022**, *27*, 919, DOI: 10.3390/molecules27030919.

(5) Schaller, D.; Šribar, D.; Noonan, T.; Deng, L.; Nguyen, T. N.; Pach, S.; Machalz, D.; Bermudez, M.; Wolber, G. Next Generation 3D Pharmacophore Modeling. *Wiley Interdiscip. Rev. Comput. Mol. Sci.* **2020**, *10*, e1468, DOI: 10.1002/wcms.1468.

(6) Sydow, D.; Wolber, G. dynophores. 2024; `https://github.com/wolberlab/dynophores`, (accessed 2024, October 28).

(7) Neale, C.; Herce, H. D.; Pomès, R.; García, A. E. Can Specific Protein-Lipid Interactions Stabilize an Active State of the Beta 2 Adrenergic Receptor? *Biophysical Journal* **2015**, *109*, 1652–1662.

(8) Sun, X.; Ågren, H.; Tu, Y. Microsecond Molecular Dynamics Simulations Provide Insight into the Allosteric Mechanism of the Gs Protein Uncoupling from the β2 Adrenergic Receptor. *The Journal of Physical Chemistry B* **2014**, *118*, 14737–14744.

(9) Ibrahim, P.; Wifling, D.; Clark, T. Universal Activation Index for Class A GPCRs. *Journal of Chemical Information and Modeling* **2019**, *59*, 3938–3945.

(10) Cheng, R. K.; Segala, E.; Robertson, N.; Deflorian, F.; Doré, A. S.; Errey, J. C.; Fiez-Vandal, C.; Marshall, F. H.; Cooke, R. M. Structures of Human A1 and A2A Adenosine Receptors with Xanthines Reveal Determinants of Selectivity. *Structure* **2017**, *25*, 1275–1285.e4.

(11) Jo, S.; Kim, T.; Iyer, V. G.; Im, W. CHARMM-GUI: A web-based graphical user interface for CHARMM. *J. Comput. Chem.* **2008**, *29*, 1859–1865.

(12) Thomas, M.; Matricon, P. G.; Gillespie, R. J.; Napiórkowska, M.; Neale, H.; Mason, J. S.; Brown, J.; Harwood, K.; Fieldhouse, C.; Swain, N. A.; Geng, T.; O'Boyle, N. M.; Deflorian, F.; Bender, A.; de Graaf, C. Identification of nanomolar adenosine A2A receptor ligands using reinforcement learning and structure-based drug design. *Nature Communications* **2025**, *16*.
